# Psychological birth trauma: A concept analysis

**DOI:** 10.3389/fpsyg.2022.1065612

**Published:** 2023-01-13

**Authors:** Xiaoqing Sun, Xuemei Fan, Shengnan Cong, Rui Wang, Lijuan Sha, Hongyan Xie, Jingjing Han, Zhu Zhu, Aixia Zhang

**Affiliations:** ^1^School of Nursing, Nanjing Medical University, Jiangsu, China; ^2^Women’s Hospital of Nanjing Medical University (Nanjing Maternity and Child Health Care Hospital), Jiangsu, China; ^3^School of Nursing, Suzhou University, Jiangsu, China

**Keywords:** birth, psychological birth trauma, concept analysis, perinatal mental health, obstetric, psychological, trauma

## Abstract

**Aim:**

To define and analyze the concept of psychological birth trauma.

**Design:**

The concept analysis method of Walker and Avant was used.

**Method:**

Eight databases (PubMed, CINAHL Complete, Cochrane Library, Web of Science, China National Knowledge Infrastructure, Wanfang, VIP Information Chinese Journal Service Platform, and Chinese BioMedicine Literature Database) were searched from inception to July 2022 for studies focused on psychological birth trauma.

**Results:**

Of the 5,372 studies identified, 44 ultimately met the inclusion criteria. The attributes identified were (1) women’s subjective feelings, (2) intertwined painful emotional experiences, (3) originating in the birth process, and (4) lasting until postpartum. Antecedents were divided into two groups: pre-existing antecedents and birth-related antecedents. Consequences were identified as negative and positive.

**Conclusion:**

Psychological birth trauma is a more complex and comprehensive concept than previously thought, and should be regarded as a separate postpartum mental health problem. This study deepens the understanding of psychological birth trauma through a comprehensive concept analysis and also puts forward some suggestions for the prevention, identification, and intervention of psychological birth trauma, which provides a basis for assisting in the identification of psychological birth trauma and provides a reference for the development of rigorous assessment tools and the design of appropriate interventions in the future. Further research is needed to update and refine this concept.

## 1. Introduction

Childbirth, a major event in a woman’s life, is of a profound and complex nature (Shorey and Wong, 2022). Not only does it involve huge physical changes, but it is also accompanied by significant psychological fluctuations (Fenech and Thomson, 2015; Shorey and Wong, 2022). Negative birth experiences can even cause terrible psychological trauma to women (Fenech and Thomson, 2015; Shorey and Wong, 2022). Studies indicated that the incidence of traumatic birth ranges from 20 to 68.6 percent in different countries (Uotila et al., 2005; Türkmen et al., 2020; Bay and Sayiner, 2021). Professor Beck used the word “ripple effect” to describe the negative impacts of psychological birth trauma (Beck, 2015). These impacts appear to be centered on the poor mental health of women themselves (Beck, 2006; Taghizadeh et al., 2013), and then expand like ripples, affecting mother-infant relationships (Taghizadeh et al., 2013; Beck and Watson, 2019), breastfeeding behavior (Beck and Watson, 2008; Fenech and Thomson, 2014), marital relationships (Taghizadeh et al., 2013; Fenech and Thomson, 2014), and future reproductive decisions (Gottvall and Waldenström, 2002; Taghizadeh et al., 2013; Holopainen et al., 2020), etc. The further impact of psychological birth trauma is associated with post-traumatic stress disorder (PTSD) (Taghizadeh et al., 2013; Türkmen et al., 2020; McKelvin et al., 2021), a widely known term. According to DSM-IV criteria, PTSD is categorized as a disorder related to trauma and stress, which is mainly manifested in four symptom clusters: re-experience, avoidance, hyperarousal, and negative cognition and mood, and these symptoms should exist for more than a month (American Psychiatric Association, 2000). These symptoms can appear directly after experiencing a traumatic event, but can also occur later in life (American Psychiatric Association, 2000). A meta-analysis suggested that 4% of postpartum women in community samples developed PTSD following a traumatic birth experience, compared with 18.5% in high-risk samples (such as women with complications of pregnancy or childbirth) (Yildiz et al., 2017). This means that more postpartum women who have experienced psychological trauma are not reaching the threshold of PTSD and are therefore unidentified, but they are struggling with the trauma.

Some studies have focused on investigating the risk factors of psychological birth trauma, and multiple factors have been found, including some objective factors, such as preterm delivery (Chabbert et al., 2021; Sommerlad et al., 2021), as well as some subjective factors, such as women feeling disrespected by healthcare professionals during birth (Zhang et al., 2020; McKelvin et al., 2021; Watson et al., 2021; Liu et al., 2022). Notably, the ongoing COVID-19 seems to have made this phenomenon more complicated. A study conducted in the United States confirmed that women who gave birth during the outbreak of COVID-19 experienced more traumatic births and subsequent mother-infant bonding problems than those who gave birth before the pandemic (Mayopoulos et al., 2021). Another study investigated the impact of unaccompanied birth caused by COVID-19-related visiting bans on mothers’ mental health, and found that mothers who gave birth unaccompanied had higher psychological distress than those who gave birth accompanied (Oddo-Sommerfeld et al., 2022).

Regrettably, not enough attention has been paid to the psychological birth trauma itself, and more attention seems to be focused on the diagnosable postpartum psychological problems, such as postpartum PTSD mentioned above. An international knowledge mapping exercise aimed at examining policies, services, and training provisions for women following traumatic birth showed that of the 18 European countries that participated, only the Netherlands has national policies on screening, treatment, and prevention of traumatic birth experiences (Thomson et al., 2021). Adding to the dilemma is the fact that there is no consistent definition, terminology, or detailed description of this concept in the literature. Instead, various terms such as “birth/childbirth trauma,” “traumatic birth/childbirth,” “traumatic birth/childbirth experience,” or “psychological birth/childbirth trauma” are used, with almost the same meaning. Additionally, widely validated tools to assess psychological birth trauma are lacking. In conclusion, there is a long way to go in the management of psychological birth trauma.

A clear concept is the first key step to fully understanding this phenomenon and the basis for theoretical development (Walker and Avant, 2019). Therefore, this study aims to provide a comprehensive analysis of the concept of psychological birth trauma, in order to clarify this definition and provide a basis for the development of rigorous assessment tools, and then provide a reference for subsequent screening and interventional research and practice. We hope this work can make some contribution to the promotion of women’s health and well-being and social development.

## 2. Materials and methods

### 2.1. Concept analysis

Concept analysis is a systematic process of developing, clarifying, and refining the phenomenon under analysis (Walker and Avant, 2019). This study adopted the concept analysis method of Walker and Avant (2019), which has been widely used in the field of nursing. It consists of eight steps intended to guide the process (Table 1). Of these, the first and second steps have been described in the introduction section of this study.

**TABLE 1:** Process of concept analysis.

Step	Process	Description	Purpose
1	Select the concept	Concept selection for analysis	Determine what is being analyzed
2	Determine the aims of the analysis	Focus on the purpose and intention of conducting the concept analysis	Determine why the analysis is needed
3	Identify concept uses	Identify as many uses of the concept as possible you can find	Understand how the concept is currently used
4	Determine defining attributes	Establish the attribute cluster most commonly associated with the concept	Analyze what features lie behind the current usage
5	Construct a model case	Use an example of the concept to demonstrate all the defining attributes of the concept	Construct an example of how the concept is currently used
6	Construct additional cases	Use examples to illustrate what the concept is not	Demonstrate that the concept is narrow enough to exclude misuse
7	Define antecedents and consequences	Antecedents: events or incidents that must occur before or be in place prior to the occurrence of the concept; Consequences: events or incidents that appear as results of the concept	Explain what happens before and after the concept is valid in usage
8	Identify empirical referents	Classes or categories of actual phenomena that by their existence demonstrate the occurrence of the concept	Check the definition of the concept for external validity

### 2.2. Data sources

A comprehensive search of PubMed, CINAHL Complete, Cochrane Library, Web of Science, China National Knowledge Infrastructure, Wanfang, VIP Information Chinese Journal Service Platform, and Chinese BioMedicine Literature Database was conducted from inception to July 2022. The following medical subject heading (MeSH) terms and text words were used: “traumatic childbirth,” “traumatic birth,” “traumatic labor,” “traumatic delivery,” “childbirth trauma,” “birth trauma,” “labor trauma,” “labor trauma,” “delivery trauma,” “psychological trauma” AND (“parturition” OR “delivery, obstetric” OR “childbirth” OR “labor” OR “birth-giving” OR “birth” OR “delivery” OR “deliver” OR “partus” OR “labor”). The search was limited to studies published in English or Chinese. Specific details of the retrieval strategy are shown in Supplementary material.

Studies that explicitly investigated or discussed psychological trauma following birth from the perspective of postpartum women were included in this concept analysis. The following studies would be excluded: (1) investigating the intervention effects of certain measures on psychological birth trauma; (2) examining physical birth trauma only; (3) only exploring psychological trauma of bystanders during the birthing process, primarily women’s partners and healthcare professionals; (4) only testing the reliability and validity of the scale. Furthermore, to improve the precision of the concept of psychological birth trauma, we excluded studies that aimed to explore postpartum PTSD and its similar themes, including postpartum post-traumatic stress symptoms and post-traumatic stress. Studies on broader topics such as negative birth experiences were also excluded.

## 3. Results

**FIGURE 1:**
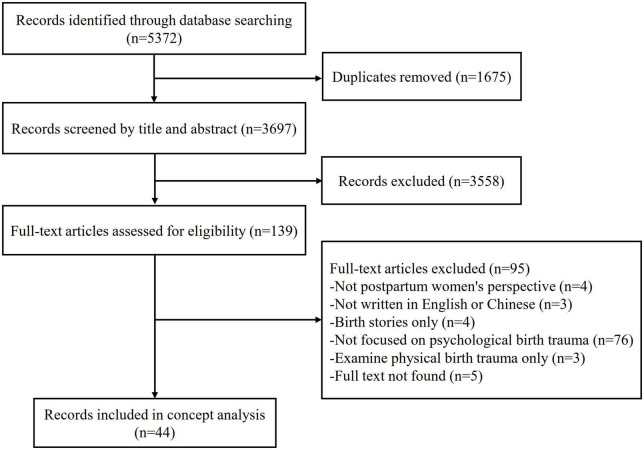
Flowchart of the study selection process of the concept analysis.

A total of 5,372 studies were identified, then 1,675 duplicated studies were excluded. After screening the titles and abstracts of the remaining studies, 3,558 studies were further excluded. After reading the full texts of 139 studies, 95 studies were excluded for a variety of reasons. Finally, a total of 44 studies were included in the concept analysis. The search process is presented in Figure 1. The specific characteristics of the included studies are presented in Table 2. The years of publication ranged from 2002 to 2022. Of the 44 studies, 10 were from the USA, nine from the UK, five from Iran, four from China, four from Australia, three from the Netherlands, three from Turkey, and one from each of the following countries: Sweden, Singapore, Germany, France, Spain, and Finland. In terms of article type, qualitative studies (*n* = 25) were the most, followed by quantitative studies (*n* = 9), and the remaining included systematic reviews (*n* = 5), mixed methods study (*n* = 1), scope review (*n* = 1), concept analysis (*n* = 1), discussion paper (*n* = 1), and middle range theory (*n* = 1).

**TABLE 2:** Studies included in concept analysis.

References	Country	Article type/Methodology	Purpose	Main results
Liu et al. (2022)	China	Meta-synthesis of qualitative research	Explore the traumatic birth experiences of women	Five synthesis results: (1) influencing factors of trauma; (2) physical and psychological trauma; (3) loss of control and participation in decision-making; (4) interpersonal relationships; (5) multiple ways to solve problems.
Shorey and Wong (2022)	Singapore	Systematic review of qualitative studies	Explore the traumatic birth experiences of parents	An overarching theme: “traumatic birth: an evolving voyage.” Three themes: (1) proceedings to traumatic birth; (2) internal and external battles; (3) muddling through to beating the odds.
Leinweber et al. (2022)	Germany	Discussion paper	Formulate a woman-centered, inclusive definition of the traumatic childbirth experience	A traumatic childbirth experience refers to a woman’s experience of interactions and/or events directly related to childbirth that caused overwhelming distressing emotions and reactions; leading to short and/or long-term negative impacts on a woman’s health and wellbeing.
Ketley et al. (2022)	UK	Qualitative	Explore how women experience post-traumatic growth following a traumatic childbirth	Three themes: (1) the total opposite to what I’d expected (participants’ struggle to understand and integrate their childbirth experience postpartum); (2) I see it a bit differently now (processes experienced in coming to a place of growth); (3) a much better place (experienced growth outcomes).
Chen et al. (2022)	China	Quantitative	Explore the relationship between women’s perceived birth trauma and risk of postpartum depression	Perceived birth trauma may be an important risk factor for postpartum depression.
Brown et al. (2022)	UK	Qualitative	Explore the process of fostering resilience after traumatic childbirth	Two main themes: (1) the feeling of powerlessness during traumatic childbirth; (2) the journey toward resilience. The powerlessness of traumatic childbirth was associated with a perceived lack of voice and abandonment by healthcare professionals.
Watson et al. (2021)	Australia	Scoping review	Explore women’s experiences of birth trauma	Three themes: (1) healthcare providers and the maternity care system; (2) women’s sense of knowing and control; (3) support.
Türkmen et al. (2021)	Turkey	Quantitative	Explore the perception of traumatic birth during pregnancy and postpartum, and the postpartum mental health outcomes of traumatic birth perception	Mean traumatic birth perception scores were significantly higher in pregnant women with high health anxiety. Traumatic birth perceptions of pregnant women who do not trust their ability and power and think that they cannot cope with labor pain were high. Postpartum women with high levels of traumatic birth perception had significantly high levels of depression, anxiety, and stress.
Molloy et al. (2021)	UK	Mixed methods	Identify maternal self-perceptions of bonding with their infants and parenting experiences following childbirth trauma	Women often described disconnection to their infants and lacking confidence in their parental decision-making. Many perceived themselves as not good enough. For some women, the trauma resulted in memory gaps of the immediate post-partum period which they found distressing, or physical recovery was so overwhelming that it impacted their capabilities to parent the way they had imagined they would. Some women developed health anxiety which resulted in an isolating experience of early parenthood.
McKelvin et al. (2021)	UK	Systematic review	Explore the psychosocial factors that may contribute to or be influenced by women’s subjective accounts of the birth	Nineteen papers were included in the review and the variables were grouped into three categories: (1) measures of labor and birth experience; (2) support and relationships; (3) psychological variables: influence and impact.
Chabbert et al. (2021)	France	Systematic review	Explore risk and protective factors affecting women’s subjective childbirth experience and birth satisfaction	The main risk factors were obstetric, such as emergency cesarean section and highly perceived labor pain, and women’s dissatisfaction with social support. The main protective factors were obstetric, including highly perceived control during labor or satisfaction regarding partner’s support.
Bay and Sayiner (2021)	Turkey	Quantitative	(1) Determine the level of traumatic birth perception and postpartum depression in women and the factors affecting them; (2) explore the relationship between traumatic birth perception and postpartum depression	The probability of experiencing postpartum depression increased four to five times in women with high or very high traumatic birth perceptions.
Zhang et al. (2020)	China	Qualitative	Explore women’s experiences of psychological birth trauma during labor and birth	Four themes: (1) How am I supposed to relieve the endless pain? (2) Can’t I be weak? (3) Am I not important? (4) What uncertainties are waiting for me?
Türkmen et al. (2020)	Turkey	Qualitative	Investigate the effect of labor comfort on traumatic birth perception, post-traumatic stress disorder, and breastfeeding after the fourth postpartum week	There was a significant relationship between physical labor comfort, transcendence, family history of labor difficulty, feelings about birth before labor begins and traumatic birth perceptions 4 weeks after birth. Physical labor comfort, psychospiritual labor comfort, transcendence, primiparity, place of residence, and traumatic birth perceptions were significantly associated with post-traumatic stress disorder 4 weeks after birth. Physical labor comfort impacted traumatic birth perceptions 3 and 6 months after birth. There was a significant relationship between high traumatic birth perception levels, high post-traumatic stress disorder prevalence, and low breastfeeding self-efficacy 3 months after birth.
Koster et al. (2020)	Netherlands	Qualitative	Investigate traumatic birth experiences of women	Three themes: (1) lack of information and consent: unilateral decision-making by maternity care professionals during intrapartum care; (2) feeling excluded: women’s maladaptive response to the one-sided decision-making healthcare professionals, leaving women feeling distant and estranged from the birth event and the experience; (3) discrepancies on an intrapersonal level: inconsistency between women’s expectations and the reality of labor and birth.
Holopainen et al. (2020)	Netherlands	Quantitative	(1) Explore which factors in the previous traumatic birth are associated with the subsequent birth experience; (2) explore the association between fear of childbirth, coping behavior during the subsequent pregnancy and subsequent childbirth experience	(1) During the subsequent pregnancy, there were 80.2% of participants reported having experienced clinically significant fear of childbirth. (2) Be prepared during subsequent pregnancy.
Dai (2019)	China	Qualitative	Explore the traumatic birth experiences of women	Eight themes: (1) excruciating pain; (2) being forgotten and ignored; (3) damaged dignity; (4) loss of children/maternal and infant separation; (5) fear; (6) desperation; (7) helplessness; (8) refusal to subsequent pregnancy.
Beck and Watson (2019)	USA	Qualitative	Explore experiences of mothers interacting with their babies after the traumatic birth	Four themes: (1) feelings of numbness and detachment; (2) crying and anger; (3) distressing cognitive changes; (4) limited outside social interactions.
Rodríguez-Almagro et al. (2019)	Spain	Qualitative	Explore women’s perceptions of traumatic birth experiences and the related factors	Five major themes: (1) birth plan compliance; (2) obstetric problems; (3) mother-infant bond; (4) emotional wounds; (5) perinatal experiences. The majority of responses mentioned feelings of being un/misinformed by healthcare personnel, being disrespected and objectified, lack of support, and various problems during birth and postpartum.
Greenfield et al. (2019)	UK	Qualitative	Explore what options women who have experienced childbirth trauma make during the prenatal period when they have another pregnancy	From the first day of pregnancy, women who have experienced childbirth trauma were focused on concerns that this birth would be a repeated traumatic experience. They were deliberately searching out and analyzing information about their choices in this pregnancy and childbirth, and making plans. They considered a range of birth choices, from elective cesarean sections to freebirth. Some women felt well supported by those around them, including care providers, partners, friends, and family. Others did not feel supported and were anticipating conflict in trying to assert their birth choices. Many early relationships with healthcare professionals were characterized by fear and mistrust.
Ghanbari-Homayi et al. (2019)	Iran	Quantitative	Assess the prevalence of a traumatic childbirth experience and identify its predictors among primiparous women	The prevalence of traumatic childbirth experience was 37%. Prenatal predictors of traumatic childbirth experience were lack of exercise during pregnancy, and intrapartum predictors were the absence of pain relief during labor and birth and fear of childbirth.
Abdollahpour and Motaghi (2019)	Iran	Qualitative	Describe traumatic childbirth experiences of mothers	Four main themes: (1) sensational and emotional experiences: anxiety, fear, sorrow, anger; (2) clinical experiences: avoidable childbirth complications, unavoidable childbirth complications; (3) legal experiences and human dignity: non-observance of the charter of patient rights, non-observance of human rights; (4) environmental experiences: lack of proper supervision, lack of proper management.
Priddis et al. (2018)	Australia	Qualitative	Explore the early parenting experiences of women who have accessed residential parenting services and consider their birth was traumatic	One overarching theme: the perfect storm of trauma. Four subthemes: (1) bringing baggage to birth; (2) trauma through a thousand cuts; (3) throwing into the pressure cooker; (4) trying to work it all out.
Murphy and Strong (2018)	UK	Qualitative	Explore childbirth trauma experiences of women	Four themes: (1) experiencing birth trauma; (2) being invisible; (3) just getting on with it; (4) making things better.
Beck (2018)	USA	Qualitative	Identify the types and frequency of mistreatment of women during childbirth in high-income countries	Six themes: (1) failure to meet professional standards of care; (2) poor rapport between women and providers; (3) verbal abuse; (4) physical abuse; (5) health system conditions/constraints; (6) stigma/discrimination.
Reed et al. (2017)	Australia	Qualitative	Understand how interpersonal factors influence women’s experience of trauma	Four themes: (1) prioritizing the care provider’s agenda; (2) disregarding embodied knowledge; (3) lies and threats; (4) violation.
Hollander et al. (2017)	Netherlands	Quantitative	Explore and quantify perceptions and experiences of women with a traumatic birth experience	Primiparous participants chose high intensity of pain/physical discomfort, long duration of delivery, and discrepancy between expectations and reality more often and fear for their own health/life, a bad outcome, and delivery went too fast less often than multiparous participants. Women primarily attribute their traumatic birth experience to lack and/or loss of control, communication issues, and practical/emotional support.
Beck and Watson (2016)	USA	Qualitative	Investigate women’s experiences of posttraumatic growth following a traumatic birth	Four themes: (1) opening oneself up to a new present; (2) achieving a new level of relationship nakedness; (3) fortifying spiritual-mindedness; (4) forging new paths.
Greenfield et al. (2016)	UK	Concept analysis and literature review	Review the literature pertaining to ‘traumatic birth’ and produce a definition of the concept	The emergence of a baby from the body of its mother, in a way which may or may not has caused physical injury. The mother finds either the events, injury, or the care she received deeply distressing or disturbing. The distress is of an enduring nature.
Taghizadeh et al. (2015)	Iran	Qualitative	Investigate the role of the environment on the psychological birth trauma	Two themes: (1) human environment: communication with mother, awareness of mother’s needs, support for mother, medical clinical competence, professional responsibility; (2) non-human environment: hospital’s physical structure, hospital’s equipment, routine care in hospital, rules governing the hospital’s environment.
Beck (2015)	USA	Middle range theory	Develop the middle range theory of traumatic childbirth	Long-term chronic consequences of traumatic childbirth for mothers include its impact on breastfeeding, subsequent childbirth, and the anniversary of childbirth trauma.
Turkstra et al. (2015)	Australia	Quantitative	Investigate the impact of high versus no childbirth distress on health-related quality of life and health-care resource utilization	High distress women had lower health-related quality of life compared to non-distressed women, more visits to general practitioners, and utilized more additional services, with no differences for infants.
Taghizadeh et al. (2014)	Iran	Qualitative	Explore the psychological birth trauma of mothers	Two themes: (1) impact of psychological birth trauma; (2) trends of psychological birth trauma. Feelings of fear, anxiety, helplessness, and sense of impending death (collapse) were reported.
Fenech and Thomson (2014)	UK	Meta-synthesis of qualitative research	Explore the psychosocial implications of traumatic childbirth on maternal well-being	Three themes: (1) consumed by demons; (2) embodied sense of loss; (3) shattered relationships.
Taghizadeh et al. (2013)	Iran	Qualitative	Explore the mothers’ response to psychological birth trauma	Three themes: (1) impact on health: physical and psychological problems; (2) changes in mother’s roles: bonding with the child, relationship with husband, social role; (3) changes decision-making ability: cesarean section request and psychological inability to have another child.
Beck (2011)	USA	Meta-ethnography	Explore traumatic birth and its aftermath	The original trigger of traumatic birth resulted in six amplifying feedback loops. Four loops were reinforcing (positive direction): (1) breastfeeding; (2) mother–infant interaction; (3) anniversary-of-birth trauma; (4) subsequent childbirth. Two loops were balancing (negative direction): (1) breastfeeding; (2) subsequent childbirth.
Elmir et al. (2010)	Australia	Meta-ethnography	Explore women’s perceptions and experiences of traumatic childbirth	Six themes: (1) feeling invisible and out of control; (2) to be treated humanely; (3) feeling trapped: the reoccurring nightmare of my childbirth experience; (4) a rollercoaster of emotions; (5) disrupted relationships; (6) strength of purpose: a way to succeed as a mother.
Beck and Watson (2010)	USA	Qualitative	Explore the meaning of women’s experiences of subsequent childbirth after a previous traumatic birth	Four themes: (1) riding the turbulent wave of panic during pregnancy; (2) strategizing: attempts to reclaim their body and complete the journey to motherhood; (3) bringing reverence to the birthing process and empowering women; (4) still elusive: the longed-for healing birth experience.
Thomson and Downe (2008)	UK	Qualitative	Investigate the lived experience of, and personal meanings attributed to, a traumatic birth	Three themes: (1) being disconnected; (2) being helpless; (3) being isolated.
Beck and Watson (2008)	USA	Qualitative	Investigate the impact of birth trauma on mothers’ breastfeeding experiences	Eight themes emerged about whether mothers’ breastfeeding attempts were promoted or impeded: (1) proving oneself as a mother: sheer determination to succeed; (2) making up for an awful arrival: atonement to the baby; (3) helping to heal mentally: time-out from the pain in one’s head; (4) just one more thing to be violated: mothers’ breasts; (5) enduring the physical pain: seeming at times an insurmountable ordeal; (6) dangerous mix: birth trauma and insufficient milk supply; (7) intruding flashbacks: stealing anticipated joy; (8) disturbing detachment: an empty affair.
Beck (2006)	USA	Qualitative	Explore the essence of mothers’ experiences regarding the anniversary of their birth trauma	Four themes: (1) the prolog: an agonizing time; (2) the actual day: a celebration of a birthday or the torment of an anniversary; (3) the epilog: a fragile state; (4) subsequent anniversaries: for better or worse.
Uotila et al. (2005)	Finland	Quantitative	(1) Investigate pre-labor attitudes and post-labor experiences of the use of vacuum extraction during delivery; (2) explore associations between traumatic labor experience and personal preparation, physiology of labor, and treatment during labor	Forty-two women (20%) regarded their birth experience as traumatic. After logistic regression analysis, only four independent risk factors showed significance: inadequate support immediately after delivery, the experience of being poorly listened to during delivery, insufficient doctor’s support during the first stage of labor, and pre-labor training classes considered inadequate.
Beck (2004)	USA	Qualitative	Explore the meaning of women’s birth trauma experiences	Four themes: (1) to care for me: was that too much to ask? (2) to communicate with me: why was this neglected? (3) to provide safe care: you betrayed my trust and I felt powerless; (4) the end justifies the means: at whose expense? at what price?
Gottvall and Waldenström (2002)	Sweden	Quantitative	Investigate the impact of the first childbirth experience on future reproduction	Women with a negative experience of their first childbirth had fewer subsequent children and a longer interval to the second baby.

### 3.1. Uses of the concept

Walker and Avant’s (2019) method involves identifying defining attributes used to describe the concept. This means that as many concept examples as possible need to be evaluated, and repeated features need to be recorded (Walker and Avant, 2019). Results of the literature showed that earlier birth trauma almost exclusively refers to maternal or neonatal physical trauma during birth, i.e., tissue and organ damage, such as maternal damaged pelvic floor function, neonatal cephalohematoma, clavicular fracture, and brachial plexus injury (Perlow et al., 1996; Meyer et al., 2000). The term further covers the long-term adverse effects of neonatal brain or skull injuries, which are usually presented in cognition (Geirsson, 1988). With the deepening of research, there is increasing evidence that childbirth can cause not only physical trauma but also psychological disturbances (Pantlen and Rohde, 2001). Professor Beck defined birth trauma in 2004 as an event that occurs during the labor and delivery process involving actual or threatened serious injury or death to the mother or her baby, in which women experience intense feelings of fear, helplessness, loss of control, and horror (Beck, 2004). This definition emphasizes the psychological experience of birth. In 2015, professor Beck integrated a series of studies on the topic of birth trauma into a whole, thereby establishing a higher and more abstract middle range theory of birth trauma, in which he clearly proposed that birth trauma can be both psychological and physical (Beck, 2015). Greenfield et al. (2016) conducted a concept analysis of traumatic birth, stating that the term can be defined as: the emergence of a baby from the mother in a way that involves events or care that cause psychological disturbance or deep distress, which may or may not involve physical injury, but leading to enduring psychological distress. A recent discussion paper developed an inclusive and woman-centered definition of the traumatic childbirth experience, which refers to a woman’s experience of interactions and/or events directly related to childbirth that caused overwhelming distressing emotions and reactions; leading to short and/or long-term negative impacts on a woman’s health and wellbeing (Leinweber et al., 2022). Apart from the three definitions above, there is no further conceptual or operational understanding.

### 3.2. Defining attributes

Defining attributes can be used to identify, understand, and differentiate a concept from other concepts (Walker and Avant, 2019). Analysis of the literature led to the identification of the most common features related to psychological birth trauma. After identifying these features, it was possible to identify key defining attributes. Thus, the attributes of psychological birth trauma are summarized as follows:

(1) Women’s subjective feelings;
(2) Intertwined painful emotional experiences;
(3) Originate in the birth process;
(4) Last until postpartum.

#### 3.2.1. Women’s subjective feeling

Women’s perception of psychological birth trauma is highly subjective (Taghizadeh et al., 2014). Each birth experience is unique to every woman, indeed, even the same events that occur during birth are perceived differently by every woman (Shorey and Wong, 2022). Studies showed that childbirth that appears normal and straightforward to healthcare professionals and is medically uneventful can be perceived as traumatic by the woman (Holopainen et al., 2020; Leinweber et al., 2022). Conversely, not all women with complications will have traumatic experiences (Leinweber et al., 2022). Notably, many studies used self-perceived psychological trauma during birth as the criteria for the recruitment and selection of research subjects (Thomson and Downe, 2008; Beck and Watson, 2019; Dai, 2019; Zhang et al., 2020). In conclusion, psychological birth trauma emphasizes the subjective feeling of women rather than objective aspects of the birth process.

#### 3.2.2. Intertwined painful emotional experiences

##### 3.2.2.1. Fear and anxiety

Faced with an unfamiliar environment and care providers, as well as an unknown birth process, women don’t know if an event will occur that threatens their own or their baby’s safety, which makes them fearful and anxious (Taghizadeh et al., 2014; Hollander et al., 2017; Dai, 2019; Holopainen et al., 2020; Zhang et al., 2020; Shorey and Wong, 2022).

##### 3.2.2.2. Helplessness and despair

During birth, women have to bear severe labor pain and face all the actual or potential risks, they do not know what to do, and the cruel reality makes them feel helpless or even despairing (Beck, 2004; Thomson and Downe, 2008; Taghizadeh et al., 2014; Dai, 2019; Zhang et al., 2020).

##### 3.2.2.3. Deprivation of dignity

Women feel verbally and physically abused and discriminated against by care providers and their privacy is violated, which deprives them of their dignity (Beck, 2015, 2018; Taghizadeh et al., 2015; Reed et al., 2017; Abdollahpour and Motaghi, 2019; Dai, 2019; Holopainen et al., 2020; Chabbert et al., 2021; Shorey and Wong, 2022).

##### 3.2.2.4. Neglected and abandoned

Women feel that they are not receiving adequate communication, explanation, emotional and practical support, and attention, they lose the power to express their thoughts and feelings as if they are a machine rather than a human, which makes them feel neglected and abandoned (Beck, 2004, 2015, 2018; Uotila et al., 2005; Elmir et al., 2010; Hollander et al., 2017; Murphy and Strong, 2018; Priddis et al., 2018; Abdollahpour and Motaghi, 2019; Dai, 2019; Rodríguez-Almagro et al., 2019; Holopainen et al., 2020; Koster et al., 2020; Zhang et al., 2020; Chabbert et al., 2021).

##### 3.2.2.5. Loss of control

Women feel deprived of decision-making and informed consent, their birth process is completely in the hands of care providers, and reality is not moving toward their expectations, which makes them feel out of control (Beck, 2004, 2015; Elmir et al., 2010; Taghizadeh et al., 2015; Hollander et al., 2017; Abdollahpour and Motaghi, 2019; Holopainen et al., 2020; Koster et al., 2020; Zhang et al., 2020; Chabbert et al., 2021; Watson et al., 2021; Liu et al., 2022).

#### 3.2.3. Originate in the birth process

Childbirth is a complex process involving medical acts and during which events that endanger the safety of the mother and baby may occur, which can be traumatic for women, especially when they believe that these events could have been avoided (Leinweber et al., 2022). In addition, the quality of women’s interactions with healthcare professionals during birth was highlighted as a major factor influencing women’s feelings about childbirth, as women used emotional language to describe their negative interaction experiences, including feeling like they were at the “bottom of the hierarch,” “persecuted,” etc. (Greenfield et al., 2019; Leinweber et al., 2022). Furthermore, psychological birth trauma may stem from events that occurred during birth that triggered women’s traumatic memories, such as sexual abuse (Watson et al., 2021).

#### 3.2.4. Last until postpartum

Persistence is a key attribute of psychological birth trauma (Greenfield et al., 2016). However, it is not entirely clear how long it lasts postpartum. In fact, many studies investigated the prevalence or effects of psychological birth trauma at 5 days (Uotila et al., 2005), 3–6 weeks (Türkmen et al., 2021), 1 month (Bay and Sayiner, 2021), and 1–4 months postpartum (Ghanbari-Homayi et al., 2019), etc., while only a few studies conducted longitudinal surveys (Türkmen et al., 2020). One study investigated the incidence of psychological birth trauma at 4 weeks, 3 months, and 6 months postpartum, unfortunately, there was no further longitudinal extension (Türkmen et al., 2020). Studies have vividly described the horrific torment of birth anniversaries that women experienced at least once (Beck, 2006, 2011, 2015). One study reported that women were formally diagnosed with postpartum PTSD 5 months to 19 years after experiencing psychological birth trauma (Beck and Watson, 2016). And even without being diagnosed with PTSD, these women are still tormented by ghosts from psychological birth trauma (Fenech and Thomson, 2014). It was indicated that there are women who still define the birth experience as psychologically traumatic even 32 years after giving birth (Taghizadeh et al., 2015).

### 3.3. Model case

A model case is designed to demonstrate all defining attributes of the concept (Walker and Avant, 2019). The model case in this study was adapted from a qualitative study aimed at investigating women’s experiences of psychological birth trauma (Beck, 2004).

Mrs. M has been having regular uterine contractions for over 5 h. A midwife asked Mrs. M to take off the pants and she would do a vaginal examination to determine the extent of cervical dilation. At this time, several students suddenly entered the room. Mrs. M tried to cover her bottom with her gown, but a midwife took her hand away from the gown. The students also performed vaginal examinations without Mrs. M’s permission, and no explanation was given afterward. Mrs. M immediately recalled the scene of being sexually assaulted when she was a child. She felt that she had been raped again, which brought her overwhelming pain. Everything seemed to be normal during the whole birth process, and finally, Mrs. M gave birth to the baby successfully, which made the midwife satisfied. All the family members surrounded the baby, leaving Mrs. M in bed alone, no one asked how she was feeling. In the days that followed, Mrs. M still felt very distressed. She was reluctant to interact with her husband and baby, refused to breastfeed, and did not trust medical staff. When the child was 3 years old, Mrs. M became pregnant again. During the pregnancy, she was surrounded by fear that this birth would repeat the previous one, and she visited a psychotherapist several times.

### 3.4. Borderline case

The authors constructed the borderline case to provide an example that embraces most of the attributes of psychological birth trauma. Mrs. N longed for a vaginal delivery and believed she could do it. Unfortunately, signs of fetal distress appeared at the beginning of the first stage of labor. Doctors told her that the prolonged labor process was dangerous to the fetus and that a cesarean section was needed as soon as possible. Mrs. N had never thought about a cesarean section, she was very scared. The doctor and midwife patiently explained to her again and gave her support and encouragement, which made Mrs. N relax. Subsequently, Mrs. N underwent an emergency cesarean section, and both mother and baby were safe. Although failed to achieve delivery vaginally as expected, Mrs. N was satisfied with the outcomes. When looking back on her birth experience, she feels supported by the healthcare providers and that she was doing the right thing.

### 3.5. Antecedents

Antecedents are those events or incidents that must occur before or be in place prior to the occurrence of the concept (Walker and Avant, 2019). Hence, in this concept analysis, antecedents refer to the precursive elements of psychological birth trauma. After reviewing the literature, considering the complexity of psychological birth trauma, antecedents are grouped according to either pre-existing or birth-related antecedents. Pre-existing antecedents refer to factors that exist prior to childbirth. These mainly include women’s demographic characteristics, personality traits, and medical and traumatic experiences. Specifically, demographic characteristics include single (Chabbert et al., 2021), low income (Bay and Sayiner, 2021), primipara (Türkmen et al., 2020; Chabbert et al., 2021), and living in the city center (Türkmen et al., 2020). Personality traits encompass insecure attachment style (Chabbert et al., 2021), high health anxiety (Türkmen et al., 2021), fear of childbirth (Ghanbari-Homayi et al., 2019; Chabbert et al., 2021), and disbelief in one’s ability to cope with labor pain (Türkmen et al., 2021). Medical and traumatic experiences encompass a history of sexual trauma (Chabbert et al., 2021), previous mental or physical health problems (Priddis et al., 2018), existing symptoms of depression or anxiety (Chabbert et al., 2021), fertility or complex pregnancy issues (Priddis et al., 2018) and a family history of labor difficulty (Türkmen et al., 2020). Furthermore, several studies reported unplanned pregnancy (Bay and Sayiner, 2021), insufficient prenatal care and training (Uotila et al., 2005; Bay and Sayiner, 2021; Chabbert et al., 2021), society stereotyped pressure on motherhood (Zhang et al., 2020), and lack of exercise during pregnancy (Ghanbari-Homayi et al., 2019) are related to psychological birth trauma.

Birth-related antecedents are key contributors to the occurrence of psychological birth trauma. These include obstetric factors and factors related to healthcare professionals. Specifically, obstetric factors encompass severe pain or physical discomfort (Uotila et al., 2005; Hollander et al., 2017; Murphy and Strong, 2018; Abdollahpour and Motaghi, 2019; Dai, 2019; Zhang et al., 2020; Chabbert et al., 2021), long duration of labor (Hollander et al., 2017; Holopainen et al., 2020; Chabbert et al., 2021), too rapid birth process (Hollander et al., 2017; Holopainen et al., 2020), unnecessary medical intervention (Reed et al., 2017; Priddis et al., 2018; Watson et al., 2021), physical restraint during birth (Shorey and Wong, 2022), cesarean section (Bay and Sayiner, 2021) or emergency cesarean section (Rodríguez-Almagro et al., 2019; Chabbert et al., 2021; Shorey and Wong, 2022), instrumental vaginal delivery (Priddis et al., 2018; Rodríguez-Almagro et al., 2019; Chabbert et al., 2021; Shorey and Wong, 2022), separation from the baby (Priddis et al., 2018; Abdollahpour and Motaghi, 2019; Dai, 2019), medical complications in infant (Holopainen et al., 2020; Liu et al., 2022) or mother (Priddis et al., 2018; Holopainen et al., 2020; Chabbert et al., 2021), dissatisfied neonatal gender (Zhang et al., 2020), preterm delivery (Chabbert et al., 2021), neonatal admission to neonatal intensive care unit (Priddis et al., 2018; Chabbert et al., 2021), neonatal death (Murphy and Strong, 2018; Dai, 2019), partner’s absence and lack of support (Holopainen et al., 2020; Chabbert et al., 2021), unpleasant birthing physical environment, equipment and rules (Taghizadeh et al., 2015; Beck, 2018; Abdollahpour and Motaghi, 2019; Watson et al., 2021; Liu et al., 2022; Shorey and Wong, 2022), and tense atmosphere during birth (Zhang et al., 2020). As for factors associated with healthcare professionals, studies have documented that the following factors are linked to psychological birth trauma: poor communication and explanation (Taghizadeh et al., 2015; Hollander et al., 2017; Beck, 2018; Priddis et al., 2018; Chabbert et al., 2021; Watson et al., 2021; Liu et al., 2022; Shorey and Wong, 2022), insufficient medical clinical competence (Taghizadeh et al., 2015; Abdollahpour and Motaghi, 2019; Rodríguez-Almagro et al., 2019), negative attitudes and words (Priddis et al., 2018), using mothers as learning resources for hospital staff (Reed et al., 2017; Watson et al., 2021), and prioritizing work agendas rather than the thoughts of women in childbirth (Reed et al., 2017; Watson et al., 2021).

### 3.6. Consequences

Consequences are those events or incidents that appear as results of the concept (Walker and Avant, 2019). Thus, in this concept analysis, consequences refer to the negative or positive effects of psychological birth trauma on women’s well-being. Firstly, there is a definite relationship between psychological birth trauma and consequences that directly affect the mental health of the mother. Following the psychological birth trauma, mothers experience heightened levels of panic, grief, anger, anxiety (Elmir et al., 2010; Fenech and Thomson, 2014), and even suicidal thoughts (Elmir et al., 2010). They are trapped in memories of the traumatic birth (Elmir et al., 2010; Shorey and Wong, 2022) and bombarded with flashbacks and nightmares (Elmir et al., 2010; Fenech and Thomson, 2014). These symptoms can last for years (Fenech and Thomson, 2014). A study described in detail their painful experiences during the subsequent birth anniversary (Beck, 2006). Additionally, these women are more likely to suffer from postpartum depression (Taghizadeh et al., 2013; Bay and Sayiner, 2021; McKelvin et al., 2021; Türkmen et al., 2021; Chen et al., 2022), anxiety (Taghizadeh et al., 2013; McKelvin et al., 2021; Türkmen et al., 2021), PTSD (Taghizadeh et al., 2013; Türkmen et al., 2020; McKelvin et al., 2021), and even psychosis (Taghizadeh et al., 2013).

Secondly, psychological birth trauma leads to changes in the mother’s roles. Several studies reported negative mother-infant interactions, with women expressing feelings of disengagement or feeble attachment to their children (Beck, 2011; Taghizadeh et al., 2013; Fenech and Thomson, 2014; Molloy et al., 2021), and feelings of incompetence as mothers (Taghizadeh et al., 2013; Fenech and Thomson, 2014; Priddis et al., 2018; Molloy et al., 2021), such as low breastfeeding self-efficacy (Türkmen et al., 2020). Mothers may experience excessive fear and anxiety about the health of their children (Molloy et al., 2021). While others described the overprotection of their children (Fenech and Thomson, 2014). Some studies reported that women experience distress while breastfeeding and therefore refuse to continue (Beck and Watson, 2008; Taghizadeh et al., 2013). Other mothers, however, insist on breastfeeding to prove that they are a mother, to help their spiritual recovery, or to atone for the baby (Beck and Watson, 2008). In addition, several studies suggested that psychological birth trauma can lead to difficulties or disruptions in couples’ emotional and sexual relationships (Taghizadeh et al., 2013; Fenech and Thomson, 2014). Moreover, some mothers display social conflicts, such as blame and aggression toward others (Taghizadeh et al., 2013; Fenech and Thomson, 2014; Watson et al., 2021), distrust and anger toward healthcare professionals (Fenech and Thomson, 2014), and a preference to remain shy and isolated (Taghizadeh et al., 2013; Fenech and Thomson, 2014).

Thirdly, psychological birth trauma affects the subsequent reproductive decisions, experiences, and coping behaviors. Women who have experienced psychological birth trauma are often fearful of future pregnancies (Fenech and Thomson, 2014; Greenfield et al., 2019; Holopainen et al., 2020; McKelvin et al., 2021), and some refuse to be pregnant again (Taghizadeh et al., 2013; Fenech and Thomson, 2014; Dai, 2019). In fact, findings suggested that women with psychological birth trauma have fewer subsequent children, as well as a longer interval to their second child (Gottvall and Waldenström, 2002). Additionally, some women who become pregnant again are surrounded by fearful thoughts that subsequent childbirth will be a repeated traumatic experience (Beck and Watson, 2010; Taghizadeh et al., 2013; Greenfield et al., 2019). And, they tend to choose a planned cesarean section (Greenfield et al., 2019; Holopainen et al., 2020), a home delivery (Holopainen et al., 2020), or a freebirth (Greenfield et al., 2019).

Fourthly, psychological birth trauma leads to more health services utilization. Women with psychological birth trauma have longer postpartum hospital stays (Turkstra et al., 2015). In addition, studies showed that they have more general practitioner visits and additional services utilization, such as psychological treatment, lactation support, child health clinic visits, and midwife home visits (Turkstra et al., 2015).

While most of the consequences of psychological birth trauma found in the literature are negative, it can also have positive outcomes. One study explored how women who have experienced psychological birth trauma rely on external and internal resources to move toward resilience (Brown et al., 2022). Moreover, several studies confirmed post-traumatic growth following psychological birth trauma (Beck and Watson, 2016; Ketley et al., 2022), including increased self-confidence and pride (Beck and Watson, 2016; Ketley et al., 2022), better relationship with partners, friends, children, and others (Beck and Watson, 2016; Ketley et al., 2022), stronger faith and a better understanding of spiritual and religious matters (Beck and Watson, 2016). Some women also established new professional and personal goals, such as completing a university degree (Beck and Watson, 2016), and actively participating in volunteer work aimed at preventing other women from psychological birth trauma (Beck and Watson, 2016; Ketley et al., 2022).

### 3.7. Empirical referents

According to Walker and Avant’s (2019) concept analysis method, the final step is to determine empirical referents of psychological birth trauma, which permits us to know how to measure or identify the defining attributes of a concept.

The Traumatic Childbirth Perception Scale (STCP) (Türkmen et al., 2021) was developed by Yalnız et al. (2016) as a self-report scale to assess women’s perception of traumatic childbirth. The STCP contains information on physical, emotional, and mental trauma associated with childbirth. It includes 13 items. Each item is scored between 0 (positive) and 10 (negative), and the total scale score ranges between 0 and 130. The total mean scores of 0–26, 27–52, 53–78, 79–104, and 105–130 correspond to very low, low, moderate, high, and very high levels of traumatic childbirth perception, respectively. In the study of Yalnız et al. (2016), the Cronbach’s alpha internal consistency coefficient of STCP was 0.895.

The Psychological Birth Trauma Questionnaire (QPBT) (Hajarian Abhari et al., 2022) was developed by Taghizadeh et al. (2013) to assess the level of psychological birth trauma. It includes 30 items and five constructs, namely: anxiety and eternal suffering of birth/labor, psychological manifestations, helplessness, collapse and sensation of death, and somatic manifestations. Each item is scored from 1 to 5 points, with a total score of 30–150 points. A higher score indicates a higher level of psychological birth trauma. The validity and reliability of QPBT have been confirmed in the study by Taghizadeh et al. (2013), the Cronbach’s alpha internal consistency coefficient was 0.949.

Notably, these two scales have not been used in studies in other countries, their English versions have not been identified yet.

### 3.8. Definition of the concept

**FIGURE 2:**
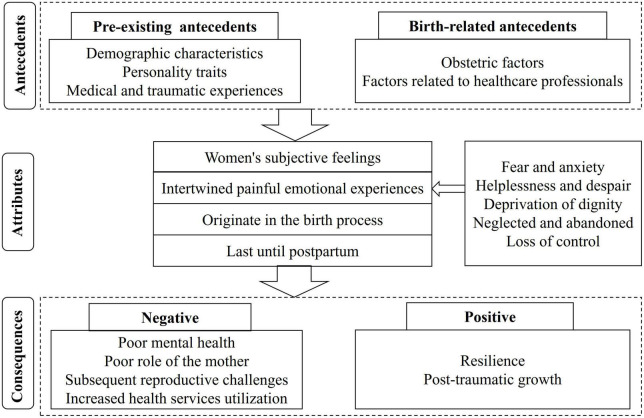
The concept of psychological birth trauma.

Based on an analysis of the literature, the concept of psychological birth trauma is clearly defined as follows:

Psychological birth trauma refers to the woman’s subjective feeling caused by events directly or indirectly related to childbirth, which is manifested as intertwined painful emotional experiences that originate in the birth process and last until postpartum. It has a wide range of negative and, in some cases, positive effects on women. The conceptual model including the antecedents, attributes, and consequences of psychological birth trauma is shown in Figure 2.

## 4. Discussion

Based on a systematic search of the literature and the method of Walker and Avant (2019), we provided a comprehensive analysis of the concept of psychological birth trauma. Four defining attributes were identified: women’s subjective feelings; intertwined painful emotional experiences; originate in the birth process and last until postpartum. Currently, maternal and neonatal safety is often regarded as the bottom line for successful and satisfying childbirth (Beck, 2004), while the subjective feeling of the mother appears to be ignored. It is worth highlighting whether women in childbirth are experiencing intertwined painful emotional experiences. Additionally, researchers and healthcare professionals should be aware that a proportion of women who have experienced psychological birth trauma are concerned by being diagnosed with postpartum PTSD, while more women who do not meet the diagnostic threshold are still in “dire straits.” Therefore, it is time to raise awareness of psychological birth trauma. Specifically, psychological birth trauma should be considered as a separate postpartum mental health problem.

Antecedents in this concept analysis suggest that women in childbirth are vulnerable to a range of pre-existing factors that may contribute to psychological trauma. Identification of these factors, including demographic characteristics, personality traits, and medical and trauma experiences can help healthcare professionals to be more vigilant and thus aid in prevention. However, the prevention of birth-related antecedents appears to be more promising. The first is to emphasize professional management of labor, such as avoiding unnecessary medical intervention, adequately relieving labor pain, and improving medical clinical competence to reduce maternal and neonatal complications. In addition, obstetric management should aim to reduce psychological birth trauma, including creating a comfortable environment and allowing the woman’s partner to accompany her during birth, etc. Furthermore, it is imperative to improve the quality of women’s interactions with healthcare professionals during birth, which may be achieved by adequately communicating and explaining what happens, listening to women, and seeking to meet their expectations. Notably, further studies are needed to systematically assess these antecedents and to determine their magnitude and interrelationships.

After examining the consequences of included studies, this concept analysis identified the profound negative effects of psychological birth trauma on women, including poor mental health, poor role of the mother, subsequent reproductive challenges, and increased health services utilization. Therefore, early identification and intervention of these women is crucial. In terms of identification, during the postpartum hospital stay, healthcare professionals are advised to be wary of symptoms that may indicate a woman has suffered psychological birth trauma, such as a dazed appearance, withdrawal, temporary amnesia, and detachment from the baby (Church and Scanlan, 2002; Beck and Watson, 2008). If a mother has experienced complex childbirth, such as maternal and neonatal complications, she should be alerted to any psychological trauma as well (Hayden, 2022). Before discharge, healthcare professionals should proactively discuss with mothers whether they perceive their childbirth to be traumatic (Beck and Watson, 2008), as new mothers may not voluntarily express their feelings about the birth experience for fear that doctors will judge their parenting or involve social services (Hayden, 2022). Try asking open-ended questions to get more information about what women might be saying (Hayden, 2022). Beck and Watson (2016) and Beck et al. (2018) recommend that healthcare professionals be wary of the metaphors women use to help describe their experiences of post-traumatic stress. In addition, at infants’ well-baby checkups and yearly physical exams, healthcare professionals are recommended to ask mothers how they are doing and how they evaluate their birth experiences (Beck, 2015). Pediatric clinicians may be in an ideal position to identify women with elevated posttraumatic stress symptoms and to make those critical referrals for mental health care (Beck, 2015).

Furthermore, a limited number of studies have discussed interventions to reduce the negative effects of psychological birth trauma. A recent systematic review examined interventions to prevent women with recent traumatic birth from developing postpartum PTSD, including debriefing, encouraging skin-to-skin contact with healthy babies directly postpartum, structured psychological interventions, expressive writing, and seeing or holding the baby after stillbirth (de Graaff et al., 2018). The results showed that there was great heterogeneity in the study characteristics, and the effectiveness of interventions was different. Possible effective interventions were encouraging skin-to-skin contact with healthy babies directly postpartum, structured psychological interventions, and expressive writing, but the evidence was insufficient (de Graaff et al., 2018). A discussion article detailed how to use an emotion-focused approach to prevent psychological birth trauma (Gökçe İsbir et al., 2022). However, this approach has not yet been applied in clinical practice. A recent study (Hajarian Abhari et al., 2022) supported the effectiveness of counseling based on Gamble’s approach, originally proposed by Gamble et al. (2005), in preventing psychological birth trauma in primiparas. The counseling approach is cost-effective, easy to implement, and can be implemented by midwives, thus it could be valuable to integrate it into healthcare programs. However, due to the huge differences among existing interventions, we cannot make wise recommendations on specific implementation programs. However, some considerations seem to be meaningful. Firstly, it is necessary to strengthen the knowledge and skills training of the interveners, which is the premise to ensure the effectiveness of the intervention. Secondly, the content, duration, frequency and timing of intervention are the key factors affecting the effectiveness, and further studies are needed to determine the optimal programs. Finally, women’s personal preferences should also be considered. Notably, the effectiveness of these interventions is mainly focused on the mental health status of women. Future studies should also examine the effectiveness of interventions on other negative effects of psychological birth trauma, such as mother-infant and marital relationships.

Reassuringly, this concept analysis found that while some women experienced some negative effects of psychological birth trauma, they gained some positive aspects that had not been mentioned in previous related concepts. Studies showed that some mothers who have experienced psychological birth trauma build resilience by using both external resources (such as faith and supportive relationships) and internal resources (such as recognizing the power of their own motherhood) (Brown et al., 2022). And, some women are able to use their inner resources to develop the resilience they need as mothers, which empowers them and allows them to experience post-traumatic growth such as an increased sense of self-worth and competence (Brown et al., 2022; Ketley et al., 2022). Thus, healthcare professionals may play an important role in facilitating this by encouraging mothers to explore their faith and use their social support networks, and informing mothers about organizations and resources that provide niche support after traumatic birth (Brown et al., 2022). In addition, healthcare professionals can provide some hope to women who have experienced psychological birth trauma by sharing the possibility of post-traumatic growth (Beck and Watson, 2016; Beck et al., 2018). Notably, the possibility of positive effects does not diminish the importance of preventing psychological birth trauma (Ketley et al., 2022).

## 5. Limitations

Some limitations exist in this concept analysis. Firstly, studies were limited to English or Chinese, which restricts the scope of the review. Secondly, the studies included in this concept analysis were mostly qualitative, and quantitative studies were lacking due to the lack of widely validated tools for assessing psychological birth trauma. Thirdly, we excluded studies on postpartum PTSD, postpartum post-traumatic stress symptoms, postpartum post-traumatic stress, and negative birth experiences, which may contain content related to psychological birth trauma. Finally, we excluded studies that explored the feelings of bystanders (especially healthcare professionals and women’s partners) who may also experience psychological trauma as a result of witnessing the birth process. We, therefore, recognize that this definition may be subject to further development and adjustment.

## 6. Conclusion

This concept analysis provides a comprehensive insight into psychological birth trauma, which is a more complex and comprehensive phenomenon than previously thought and should be considered as a separate postpartum mental health problem. Given the high incidence and far-reaching effects of psychological birth trauma, its prevention, identification and intervention are crucial, but relevant studies are insufficient and need to be further explored. This study provides a starting point for future theory and research, and provides researchers and healthcare professionals with information that can serve as the foundation for assisting in the identification of psychological birth trauma, and as the reference for developing rigorous assessment tools as well as designing appropriate interventions. In addition, further research is needed to expand the definition of psychological birth trauma from the perspective of bystanders during birth. We expect that this concept will continue to be updated and refined as knowledge on the subject develops.

## Author contributions

XS, XF, SC, RW, LS, HX, JH, ZZ, and AZ designed the study, critically revised the manuscript, and approved the final version. XS and XF conducted the literature retrieval, literature selection, data extraction, data analysis, and drafted the manuscript. All authors contributed to the article and approved the submitted version.

## References

[B1] Abdollahpour S. Motaghi Z. (2019). Lived traumatic childbirth experiences of newly delivered mothers admitted to the postpartum ward: A phenomenological study. *J. Caring Sci.* 8 23–31. 10.15171/jcs.2019.004 30915310 PMC6428162

[B2] American Psychiatric Association (2000). *Diagnostic and statistical manual of mental disorders*, 4th Edn. Washington, DC: American Psychiatric Pub.

[B3] Bay F. Sayiner F. D. (2021). Perception of traumatic childbirth of women and its relationship with postpartum depression. *Women Health* 61 479–489. 10.1080/03630242.2021.1927287 33980127

[B4] Beck C. T. (2004). Birth trauma: In the eye of the beholder. *Nurs. Res.* 53 28–35. 10.1097/00006199-200401000-00005 14726774

[B5] Beck C. T. (2006). The anniversary of birth trauma: Failure to rescue. *Nurs. Res.* 55 381–390. 10.1097/00006199-200611000-00002 17133145

[B6] Beck C. T. (2011). A metaethnography of traumatic childbirth and its aftermath: Amplifying causal looping. *Qual. Health Res.* 21 301–311. 21131566 10.1177/1049732310390698

[B7] Beck C. T. (2015). Middle range theory of traumatic childbirth: The ever-widening ripple effect. *Glob. Qual. Nurs. Res.* 2:2333393615575313. 10.1177/2333393615575313 28462301 PMC5342633

[B8] Beck C. T. (2018). A secondary analysis of mistreatment of women during childbirth in health care facilities. *J. Obstetr. Gynecol. Neonatal Nurs.* 47 94–104. 10.1016/j.jogn.2016.08.015 28453947

[B9] Beck C. T. Watson S. (2008). Impact of birth trauma on breast-feeding: A tale of two pathways. *Nurs. Res.* 57 228–236. 10.1097/01.NNR.0000313494.87282.90 18641491

[B10] Beck C. T. Watson S. (2010). Subsequent childbirth after a previous traumatic birth. *Nurs. Res.* 59 241–249. 10.1097/NNR.0b013e3181e501fd 20585221

[B11] Beck C. T. Watson S. (2016). Posttraumatic growth after birth trauma: “I was broken. *MCN Am. J. Matern. Child Nurs.* 41 264–271. 10.1097/NMC.0000000000000259 27276105

[B12] Beck C. T. Watson S. (2019). Mothers’ experiences interacting with infants after traumatic childbirth. *MCN Am. J. Matern. Child Nurs.* 44 338–344. 10.1097/NMC.0000000000000565 31469669

[B13] Beck C. T. Watson S. Gable R. K. (2018). Traumatic childbirth and its aftermath: Is there anything positive? *J. Perinat. Educ.* 27 175–184. 10.1891/1058-1243.27.3.175 30364308 PMC6193358

[B14] Brown A. Nielsen J. D. J. Russo K. Ayers S. Webb R. (2022). The journey towards resilience following a traumatic birth: A grounded theory. *Midwifery* 104:103204. 10.1016/j.midw.2021.103204 34839226

[B15] Chabbert M. Panagiotou D. Wendland J. (2021). Predictive factors of women’s subjective perception of childbirth experience: A systematic review of the literature. *J. Reprod. Infant Psychol.* 39 43–66. 10.1080/02646838.2020.1748582 32475156

[B16] Chen Y. Ismail F. Xiong Z. Li M. Chen I. Wen S. W. (2022). Association between perceived birth trauma and postpartum depression: A prospective cohort study in China. *Int. J. Gynaecol. Obstetr.* 157 598–603. 10.1002/ijgo.13845 34324705

[B17] Church S. Scanlan M. (2002). Post-traumatic stress disorder after childbirth. Do midwives have a preventative role? *Practis. Midwife* 5 10–13. 12099126

[B18] Dai L. (2019). *Research on the experience of birth trauma and its influencing factors.* Ph.D. thesis. Wuhan: Huazhong University of Science and Technology.

[B19] de Graaff L. F. Honig A. van Pampus M. G. Stramrood C. A. I. (2018). Preventing post-traumatic stress disorder following childbirth and traumatic birth experiences: A systematic review. *Acta Obstetr. Gynecol. Scand.* 97 648–656. 10.1111/aogs.13291 29336486

[B20] Elmir R. Schmied V. Wilkes L. Jackson D. (2010). Women’s perceptions and experiences of a traumatic birth: A meta-ethnography. *J. Adv. Nurs.* 66 2142–2153. 10.1111/j.1365-2648.2010.05391.x 20636467

[B21] Fenech G. Thomson G. (2014). Tormented by ghosts from their past’: A meta-synthesis to explore the psychosocial implications of a traumatic birth on maternal well-being. *Midwifery* 30 185–193. 10.1016/j.midw.2013.12.004 24411664

[B22] Fenech G. Thomson G. (2015). Defence against trauma: Women’s use of defence mechanisms following childbirth-related trauma. *J. Reprod. Infant Psychol.* 33 268–281. 10.1080/02646838.2015.1030731

[B23] Gamble J. Creedy D. Moyle W. Webster J. McAllister M. Dickson P. (2005). Effectiveness of a counseling intervention after a traumatic childbirth: A randomized controlled trial. *Birth* 32 11–19. 10.1111/j.0730-7659.2005.00340.x 15725200

[B24] Geirsson R. T. (1988). Birth trauma and brain damage. *Baillieres Clin. Obstetr. Gynaecol.* 2 195–212. 10.1016/s0950-3552(88)80072-5 3046800

[B25] Ghanbari-Homayi S. Fardiazar Z. Meedya S. Mohammad-Alizadeh-Charandabi S. Asghari-Jafarabadi M. Mohammadi E. (2019). Predictors of traumatic birth experience among a group of Iranian primipara women: A cross sectional study. *BMC Pregnancy Childbirth* 19:182. 10.1186/s12884-019-2333-4 31117987 PMC6532129

[B26] Gökçe İsbir G. Yılmaz M. Thomson G. (2022). Using an emotion-focused approach in preventing psychological birth trauma. *Perspect. Psychiatr. Care* 58 1170–1176. 10.1111/ppc.12867 34047362

[B27] Gottvall K. Waldenström U. (2002). Does a traumatic birth experience have an impact on future reproduction? *BJOG Int. J. Obstetr. Gynaecol.* 109 254–260. 10.1111/j.1471-0528.2002.01200.x 11950179

[B28] Greenfield M. Jomeen J. Glover L. (2016). What is traumatic birth? A concept analysis and literature review. *Br. J. Midwifery* 24 254–267. 10.12968/bjom.2016.24.4.254

[B29] Greenfield M. Jomeen J. Glover L. (2019). ”It can’t be like last time”–choices made in early pregnancy by women who have previously experienced a traumatic birth. *Front. Psychol.* 10:56. 10.3389/fpsyg.2019.00056 30740076 PMC6355667

[B30] Hajarian Abhari Z. Karimi F. Z. Taghizdeh Z. Mazloum S. R. Asghari Nekah S. M. (2022). Effects of counseling based on Gamble’s approach on psychological birth trauma in primiparous women: A randomized clinical trial. *J. Matern. Fetal Neonatal Med.* 35 668–676. 10.1080/14767058.2020.1730799 32089025

[B31] Hayden E. (2022). It felt like my birth trauma had been forgotten. *BMJ (Clin. Res. Ed.)* 377:o1006. 10.1136/bmj.o1006 35537753

[B32] Hollander M. Hastenberg E. Dillen J. Pampus M. Miranda E. Stramrood C. (2017). Preventing traumatic childbirth experiences: 2192 women’s perceptions and views. *Arch. Womens Mental Health* 20 515–523. 10.1007/s00737-017-0729-6 28553692 PMC5509770

[B33] Holopainen A. Stramrood C. van Pampus M. G. Hollander M. Schuengel C. (2020). Subsequent childbirth after previous traumatic birth experience: Women’s choices and evaluations. *Br. J. Midwifery* 28 488–496. 10.12968/bjom.2020.28.8.488

[B34] Ketley R. Darwin Z. Masterson C. McGowan L. (2022). Women’s experience of post-traumatic growth following a traumatic birth: An interpretive phenomenological analysis. *J. Reprod. Infant Psychol.* 27 1–12. 10.1080/02646838.2022.2070608 35475719

[B35] Koster D. Romijn C. Sakko E. Stam C. Steenhuis N. de Vries D. (2020). Traumatic childbirth experiences: Practice-based implications for maternity care professionals from the woman’s perspective. *Scand. J. Caring Sci.* 34 792–799. 10.1111/scs.12786 31657049

[B36] Leinweber J. Fontein-Kuipers Y. Thomson G. Karlsdottir S. I. Nilsson C. Ekström-Bergström A. (2022). Developing a woman-centered, inclusive definition of traumatic childbirth experiences: A discussion paper. *Birth* 49 687–696. 10.1111/birt.12634 35403241

[B37] Liu J. Qiao J. H. Zhou S. J. Lv J. Liu R. S. Wen H. (2022). Meta synthesis of qualitative research on women’s real experience of childbirth trauma. *Chin. J. Modern Nurs.* 28 2–8. 10.3760/cma.j.cn115682-20210813-03598 30704229

[B38] Mayopoulos G. A. Ein-Dor T. Dishy G. A. Nandru R. Chan S. J. Hanley L. E. (2021). COVID-19 is associated with traumatic childbirth and subsequent mother-infant bonding problems. *J. Affect. Disord.* 282 122–125. 10.1016/j.jad.2020.12.101 33412491 PMC7889625

[B39] McKelvin G. Thomson G. Downe S. (2021). The childbirth experience: A systematic review of predictors and outcomes. *Women Birth* 34 407–416. 10.1016/j.wombi.2020.09.021 33039281

[B40] Meyer S. Hohlfeld P. Achtari C. Russolo A. De Grandi P. (2000). Birth trauma: Short and long term effects of forceps delivery compared with spontaneous delivery on various pelvic floor parameters. *BJOG* 107 1360–1365. 10.1111/j.1471-0528.2000.tb11648.x 11117762

[B41] Molloy E. Biggerstaff D. L. Sidebotham P. (2021). A phenomenological exploration of parenting after birth trauma: Mothers perceptions of the first year. *Women birth* 34 278–287. 10.1016/j.wombi.2020.03.004 32303461

[B42] Murphy H. Strong J. (2018). Just another ordinary bad birth? A narrative analysis of first time mothers’ traumatic birth experiences. *Health Care Women Int.* 39 619–643. 10.1080/07399332.2018.1442838 29474791

[B43] Oddo-Sommerfeld S. Schermelleh-Engel K. Konopka M. La Rosa V. L. Louwen F. Sommerlad S. (2022). Giving birth alone due to COVID-19-related hospital restrictions compared to accompanied birth: Psychological distress in women with caesarean section or vaginal birth - a cross-sectional study. *J. Perinat. Med.* 50 539–548. 10.1515/jpm-2021-0368 35357796

[B44] Pantlen A. Rohde A. (2001). Psychologic effects of traumatic live deliveries. *Zentralbl. Gynakol.* 123 42–47. 10.1055/s-2001-12025 11385911

[B45] Perlow J. H. Wigton T. Hart J. Strassner H. T. Nageotte M. P. Wolk B. M. (1996). Birth trauma. A five-year review of incidence and associated perinatal factors. *J. Reprod. Med.* 41 754–760. 8913978

[B46] Priddis H. S. Keedle H. Dahlen H. (2018). The perfect storm of trauma: The experiences of women who have experienced birth trauma and subsequently accessed residential parenting services in Australia. *Women Birth* 31 17–24. 10.1016/j.wombi.2017.06.007 28666701

[B47] Reed R. Sharman R. Inglis C. (2017). Women’s descriptions of childbirth trauma relating to care provider actions and interactions. *BMC Pregnancy Childbirth* 17:21. 10.1186/s12884-016-1197-0 28068932 PMC5223347

[B48] Rodríguez-Almagro J. Hernández-Martínez A. Rodríguez-Almagro D. Quirós-García J. M. Martínez-Galiano J. M. Gómez-Salgado J. (2019). Women’s perceptions of living a traumatic childbirth experience and factors related to a birth experience. *Int. J. Environ. Res. Public Health* 16:1654. 10.3390/ijerph16091654 31085980 PMC6539242

[B49] Shorey S. Wong P. Z. E. (2022). Traumatic childbirth experiences of new parents: A meta-synthesis. *Trauma Violence Abuse* 23 748–763. 10.1177/1524838020977161 33256544

[B50] Sommerlad S. Schermelleh-Engel K. La Rosa V. L. Louwen F. Oddo-Sommerfeld S. (2021). Trait anxiety and unplanned delivery mode enhance the risk for childbirth-related post-traumatic stress disorder symptoms in women with and without risk of preterm birth: A multi sample path analysis. *PLoS One* 16:e0256681. 10.1371/journal.pone.0256681 34464408 PMC8407573

[B51] Taghizadeh Z. Arbabi M. Kazemnejad A. Irajpour A. Lopez V. (2015). Iranian mothers’ perceptions of the impact of the environment on psychological birth trauma: A qualitative study. *Int. J. Nurs. Pract.* 21(Suppl. 2) 58–66. 10.1111/ijn.12286 24758150

[B52] Taghizadeh Z. Irajpour A. Arbabi M. (2013). Mothers’ response to psychological birth trauma: A qualitative study. *Iran. Red Crescent Med. J.* 15:e10572. 10.5812/ircmj.10572 24693361 PMC3950773

[B53] Taghizadeh Z. Irajpour A. Nedjat S. Arbabi M. Lopez V. (2014). Iranian mothers’ perception of the psychological birth trauma: A qualitative study. *Iran. J. Psychiatry* 9 31–36. 25561946 PMC4277605

[B54] Thomson G. Downe S. (2008). Widening the trauma discourse: The link between childbirth and experiences of abuse. *J. Psychosom. Obstetr. Gynaecol.* 29 268–273. 10.1080/01674820802545453 19065396

[B55] Thomson G. Diop M. Q. Stuijfzand S. Horsch A. (2021). Policy, service, and training provision for women following a traumatic birth: An international knowledge mapping exercise. *BMC Health Serv. Res.* 21:1206. 10.1186/s12913-021-07238-x 34742293 PMC8571982

[B56] Türkmen H. Yalniz Dilcen H. Akin B. (2020). The effect of labor comfort on traumatic childbirth perception, post-traumatic stress disorder, and breastfeeding. *Breastfeed. Med.* 15 779–788. 10.1089/bfm.2020.0138 32896164

[B57] Türkmen H. Yalniz Dilcen H. Ozcoban F. A. (2021). Traumatic childbirth perception during pregnancy and the postpartum period and its postnatal mental health outcomes: A prospective longitudinal study. *J. Reprod. Infant Psychol.* 39 422–434. 10.1080/02646838.2020.1792429 32673072

[B58] Turkstra E. Creedy D. K. Fenwick J. Buist A. Scuffham P. A. Gamble J. (2015). Health services utilization of women following a traumatic birth. *Arch. Womens Mental Health* 18 829–832. 10.1007/s00737-014-0495-7 25577338

[B59] Uotila J. T. Taurio K. Salmelin R. Kirkinen P. (2005). Traumatic experience with vacuum extraction–influence of personal preparation, physiology, and treatment during labor. *J. Perinat. Med.* 33 373–378. 10.1515/jpm.2005.068 16238530

[B60] Walker L. O. Avant K. C. (2019). *Strategies for theory construction in nursing*, 6th Edn. Upper Saddle River, NJ: Pearson.

[B61] Watson K. White C. Hall H. Hewitt A. (2021). Women’s experiences of birth trauma: A scoping review. *Women and Birth* 34 417–424. 33020046 10.1016/j.wombi.2020.09.016

[B62] Yalnız H. Canan F. Genç R. E. Kuloğlu M. M. Gecici Ö (2016). Development of a scale of traumatic childbirth perception. *Turk. J. Med. Sci*. 8, 81–88. 10.5505/ttd.2016.40427

[B63] Yildiz P. D. Ayers S. Phillips L. (2017). The prevalence of posttraumatic stress disorder in pregnancy and after birth: A systematic review and meta-analysis. *J. Affect. Disord.* 208 634–645. 10.1016/j.jad.2016.10.009 27865585

[B64] Zhang K. Dai L. Wu M. Zeng T. Yuan M. Chen Y. (2020). Women’s experience of psychological birth trauma in China: A qualitative study. *BMC Pregnancy Childbirth* 20:651. 10.1186/s12884-020-03342-8 33109113 PMC7590597

