# Supplementary material for: Psychological birth trauma: A concept analysis
Source: Front Psychol. 2023 Jan 13;13:1065612. doi: 10.3389/fpsyg.2022.1065612 (PMC9880163; doi:10.3389/fpsyg.2022.1065612)
Supplement: Supplementary file 1 [file Table_1.DOCX]

**Search strategies for all database**

| **Database** | **Search strategy** | |
| --- | --- | --- |
| **Pubmed** | #1 | "psychological trauma"[MeSH Terms] |
|  | #2 | "psychological trauma"[Title/Abstract]) |
|  | #3 | #1 or #2 |
|  | #4 | "parturition"[MeSH Terms] OR "delivery, obstetric"[MeSH Terms] |
|  | #5 | ("parturition"[Title/Abstract] OR "childbirth"[Title/Abstract] OR "labor"[Title/Abstract] OR "birth-giving"[Title/Abstract] OR "birth"[Title/Abstract] OR "delivery"[Title/Abstract] OR "deliver"[Title/Abstract] OR "partus"[Title/Abstract] OR "labour"[Title/Abstract] |
|  | #6 | #4 or #5 |
|  | #7 | #3 AND #6 |
|  | #8 | "traumatic childbirth"[Title/Abstract] OR "traumatic labor"[Title/Abstract] OR "traumatic birth"[Title/Abstract] OR "traumatic delivery"[Title/Abstract] OR ("childbirth trauma"[Title/Abstract] OR "labor trauma"[Title/Abstract] OR "birth trauma"[Title/Abstract] OR "delivery trauma"[Title/Abstract] OR "labour trauma"[Title/Abstract]) |
|  | #9 | #7 or #8 |
| **CINAHL Complete** | #1 | SU childbirth OR SU labor OR AB childbirth OR AB labor OR AB parturition OR AB birth-giving OR AB birth OR AB delivery OR AB deliver OR AB partus OR AB labour |
|  | #2 | SU Psychological Trauma OR AB Psychological Trauma |
|  | #3 | #1AND #2 |
|  | #4 | AB "traumatic childbirth" OR AB "traumatic labor" OR AB "traumatic birth" OR AB "traumatic delivery" OR AB "traumatic labour" OR AB "traumatic parturition" OR AB "traumatic birth-giving" OR AB "traumatic deliver" OR AB "traumatic partus" |
|  | #5 | AB "childbirth trauma" OR AB "labor trauma" OR AB "birth trauma" OR AB "delivery trauma" OR AB "labour trauma" OR AB "parturition trauma" OR AB "birth-giving trauma" OR AB "deliver trauma" OR AB "partus trauma" |
|  | #6 | #3 OR #4 OR #5 |
| **Cochrane Library** | #1 | (parturition):ti,ab,kw OR (delivery):ti,ab,kw OR (birth):ti,ab,kw OR (childbirth):ti,ab,kw OR (deliver):ti,ab,kw OR (partus):ti,ab,kw OR (labour):ti,ab,kw OR (labor):ti,ab,kw OR (birth-giving):ti,ab,kw |
|  | #2 | MeSH descriptor: [Parturition] explode all trees |
|  | #3 | #1 OR #2 |
|  | #4 | ("Psychological Trauma"):ti,ab,kw |
|  | #5 | MeSH descriptor: [Psychological Trauma] explode all trees |
|  | #6 | #4 OR #5 |
|  | #7 | #3 AND #6 |
|  | #8 | ("traumatic childbirth"):ti,ab,kw OR ("traumatic labor"):ti,ab,kw OR ("traumatic birth"):ti,ab,kw OR ("traumatic delivery"):ti,ab,kw OR ("traumatic labour"):ti,ab,kw OR ("traumatic parturition"):ti,ab,kw OR ("traumatic birth-giving"):ti,ab,kw OR ("traumatic deliver"):ti,ab,kw OR ("traumatic partus"):ti,ab,kw |
|  | #9 | ("childbirth trauma"):ti,ab,kw OR ("labor trauma"):ti,ab,kw OR ("birth trauma"):ti,ab,kw OR ("delivery trauma"):ti,ab,kw OR ("labour trauma"):ti,ab,kw OR ("parturition trauma"):ti,ab,kw OR ("birth-giving trauma"):ti,ab,kw OR ("deliver trauma"):ti,ab,kw OR ("partus trauma"):ti,ab,kw |
|  | #10 | #7 OR #8 OR #9 |
| **Web of Science** | #1 | AB=("psychological trauma") |
|  | #2 | ((((((((AB=(parturition)) OR AB=(childbirth)) OR AB=(birth)) OR AB=(delivery)) OR AB=(deliver)) OR AB=(labor)) OR AB=(labour)) OR AB=(birth-giving)) OR AB=(partus) |
|  | #3 | #1 AND #2 |
|  | #4 | ((((((((AB=("traumatic childbirth")) OR AB=("traumatic labor")) OR AB=("traumatic birth")) OR AB=("traumatic delivery")) OR AB=("traumatic labour")) OR AB=("traumatic parturition")) OR AB=("traumatic birth-giving")) OR AB=("traumatic deliver")) OR AB=("traumatic partus") |
|  | #5 | ((((((((AB=("childbirth trauma")) OR AB=("labor trauma")) OR AB=("birth trauma")) OR AB=("delivery trauma")) OR AB=("labour trauma")) OR AB=("parturition trauma")) OR AB=("birth-giving trauma")) OR AB=("deliver trauma")) OR AB=("partus trauma") |
|  | #6 | #3 OR #4 OR #5 |
| **China National Knowledge Infrastructure** | #1 | (AB=分娩创伤 or SU=分娩创伤) OR (AB=创伤性分娩 or SU=创伤性分娩) OR （(AB=心理创伤 or SU=心理创伤) AND (AB=分娩 or SU=分娩) ） |
| **Wangfang** | #1 | (摘要=分娩创伤 or 主题=分娩创伤) OR (摘要=创伤性分娩 or主题=创伤性分娩) OR （(摘要=心理创伤 or主题=心理创伤) AND (摘要=分娩 or主题=分娩) ） |
| **VIP** **Information Chinese Journal Service Platform** | #1 | (摘要=分娩创伤) OR (摘要=创伤性分娩) OR (摘要=心理创伤 AND摘要=分娩) |
| **Chinese BioMedicine Literature Database** | #1 | ("创伤性分娩"[摘要]) OR ("分娩创伤"[摘要]) OR ("心理创伤"[摘要] AND "分娩"[摘要]) |
